# Synthesis of unequally-spaced arrays using the fractional Fourier series

**DOI:** 10.1038/s41598-022-23473-6

**Published:** 2022-11-07

**Authors:** Mahdi Boozari, Mohammad Khalaj-Amirhosseini

**Affiliations:** 1 Electrical Dept., Ferdwosi University of Mashhad, Mashhad, Iran; 2 School of Electrical Engineering, Iran University of Science and Technology, Tehran, Iran

**Keywords:** Engineering, Electrical and electronic engineering

## Abstract

This paper introduces an algebraic method to synthesize the radiation pattern of unequally-spaced arrays. In this method, first, the characteristic matrix, containing the sampled data of the desired pattern, is established. Then, the fractional Fourier series is calculated using the distinct eigenvalues and eigenvectors of the characteristic matrix. In the following, the location of the array elements is estimated using the eigenvalues by considering the mutual coupling effect. The magnitude and phase of the excitation currents are computed using the least square method. Also, it is proved that the number of elements can be reduced using the eigenvalue decomposition and differential spectrum method. Several important patterns are investigated to verify the performance of the proposed method, and then a comprehensive discussion is expressed for all cases. It is shown that using the proposed method, a greater average spacing is achieved which allows bettering mitigate the phenomenon of mutual coupling. Furthermore, the various results show that the proposed method offers a better approximation of the desired array factor especially for beam-shaped patterns. This method also has a noble ability to reduce the number of array elements compared to a generic reference array, while still retaining a good ability to approximate the desired pattern fairly faithfully.

## Introduction

An antenna array can replace a single antenna to achieve high gain, side lobe levels (SLLs) controlling, and capability of the spatial scan. These advantages make them suitable for many applications in electronic countermeasures, multitask communication systems, and radar systems. The radiation pattern of an antenna array is usually synthesized by determining both the magnitude and phase of the composing elements^1–4^.

An unequally spaced array has many advantages in comparison to those of array, including low profile, simple beam forming structure, low cost^5,6^. The magnitude and phase of the array elements, as well as the location of each element, are unknown in unequally spaced arrays. Designing the unequally spaced arrays with the minimum number of elements is a complex problem compared to those of the equally spaced arrays, especially in satellite communication in which a low weight antenna is required^7,8^.

There are some analytical, probabilistic and density-tapering methods for unequally-spaced arrays. In^9^, several probabilistic features of the large arrays with randomly spaced elements have been investigated. It is shown that the necessary number of elements is related to the chosen SLL. In^10^, the statistical density-tapered array has been studied. It is shown that the proposed method is a suitable technique to accomplish a radiation patterns with good SLL without the requirement of an amplitude tapering. A method based on the Poisson’s sum formula and source position function is introduced in^11^. In this technique, the desired pattern in converted into a series of integrals by choosing a suitable transformation. This technique can be used to determine an appropriate amplitude, phase and location of array elements. In^12^, space tapering method is used to reduce the number of array elements of an unequally-spaced array. In this technique, the conventional amplitude distribution array is replicated by changing the spacing of equally excited elements to predict the gain, beam-width and SLL. A method established based on the sampling theorem for band-limited functions is introduced in^13^. Using it, the probability of SLL of any array is determined for a given probability density of element locations.

The algorithm-based methods, including genetic algorithm (GA)^14^ and differential evolution algorithm (DEA)^15^, have been widely used to find the unknown parameters of these types of arrays. Although these methods have been discussed in the literature, they don’t provide an obvious relationship between the prescribed pattern and the unknown parameters. Furthermore, the algorithm-based techniques are time-consuming.

The other methods include the orthogonal techniques based on the ultra-spherical polynomials^16,17^, iterative FFT via virtual active element pattern expansion^18^, and matrix pencil method (MPM)^19^, eigenvector decomposition^20^. Despite exciting aspects of these methods, they are typically complex and don’t have the reduction capability of the number of array elements. Additionally, in most of them, the mutual coupling is ignored. Hence, the application of these methods is limited or should be used for particular problems.

In this work, a different method is introduced to synthesize the radiation pattern of the unequally-spaced arrays. This method tries to design an array with a radiation pattern as close to the corresponding prescribed array factor. First, the characteristic matrix is established using the samples of the desired array factor. The sampling step is an important aspect of synthesizing procedure. Hence, the Nyquist sampling theorem is used in this step. Next, the reconstructed array factor is rewritten as the fractional Fourier series by the concept of eigenvalue. By comparing the fractional Fourier series and the array factor, it is seen that the location of elements can be determined from the phase of eigenvalues. After specifying the location of elements, the least square method is used to determine the excitation currents. The differential spectrum method is employed to reduce the number of array elements. Additionally, a few case studies are examined to verify the performance of the proposed method. The results show that using the introduced technique, a greater average spacing is achieved which allows bettering mitigate the phenomenon of mutual coupling. The accuracy of the proposed method is noteworthy than especially for beam-shaped patterns. This method also has an acceptable ability to reduce the number of array elements.

## Mathematical formulation

The array factor of an unequally spaced linear array oriented along *z*-axis direction can be expressed as Eq. (1), in which, *z*_*n*_ = *kd*_*n*_ and *I*_*n*_, *d*_*n*_, *N* and *k* show the complex excitation current and location of the *n*th element, the total number of array elements and the wave number defined by 2π/λ (λ is wavelength), respectively, and *u* = cos*θ*^21^.

1 $$ F = \sum\limits_{n = 1}^{N} {I_{n} \exp \left( {juz_{n} } \right)} $$

The synthesis process of an unequally spaced array is a non-linear problem with 2* N* unknowns. It is assumed that the length of the array is *L*. So, according to the Nyquist sampling theorem, the desired array factor can be reconstructed using the uniform sampling step ∆ as.

2 $$ \Delta \le {\lambda \mathord{\left/ {\vphantom {\lambda {\left( {2L} \right)}}} \right. \kern-\nulldelimiterspace} {\left( {2L} \right)}} $$

By assuming ∆≈1/(2*L*), only 2* M* + 1 samples are sufficient for reconstructing the prescribed array factor. Therefore, the location of samples over the interval − 1 ≤ *u* ≤  + 1 is as follows.

3 $$ u_{m} \simeq m\Delta = \frac{m\lambda }{{2L}}, \, m = - M, \, ..., \, 0, \, ..., \, M $$

The vector **V**, holding the samples of the desired pattern, can be written as.

4 $$ {\mathbf{V}} = \left[ {F_{d} \left( {{{m\lambda } \mathord{\left/ {\vphantom {{m\lambda } {2L}}} \right. \kern-\nulldelimiterspace} {2L}}} \right)} \right]_{{1 \times \left( {2M + 1} \right)}} ,m = - M, \, ..., \, 0, \, ..., \, M $$

where *F*_*d*_ is the desired array factor. In the following, two sample matrices **HF**, **HL** can be organized using vector **V** ^20^.

5 $$ {\mathbf{HF}} = \left[ {\begin{array}{*{20}c} {V_{2} } & {V_{3} } & \cdots & {V_{M + 1} } \\ {V_{3} } & {V_{4} } & \cdots & {V_{M + 2} } \\ \vdots & \vdots & \ddots & \vdots \\ {V_{M + 2} } & {V_{M + 3} } & \cdots & {V_{2M + 1} } \\ \end{array} } \right]_{{\left( {M + 1} \right) \times M}} $$

6 $$ {\mathbf{HL}} = \left[ {\begin{array}{*{20}c} {V_{1} } & {V_{2} } & \cdots & {V_{M} } \\ {V_{2} } & {V_{3} } & \cdots & {V_{M + 1} } \\ \vdots & \vdots & \ddots & \vdots \\ {V_{M + 1} } & {V_{M + 2} } & \cdots & {V_{2M} } \\ \end{array} } \right]_{{\left( {M + 1} \right) \times M}} $$

Two matrices **HF**, **HL** are combined into a single matrix **H** using the following equation^22^.

7 $$ {\mathbf{H}} = \left( {{\mathbf{HL}}^{T} {\mathbf{HL}}} \right)^{ - 1} {\mathbf{HL}}^{T} {\mathbf{HF}} $$

in which **H** is the characteristic matrix of the under-studying problem. From the matrix algebra, it can be shown that the fractional Fourier series of the problem can be determined using the distinct eigenvalues and eigenvectors of the characteristic matrix^20,22^.

8 $$ F_{r} = \sum\limits_{n = 1}^{N} {c_{n} Y_{n} \exp \left( {\zeta_{n} u} \right)} $$

in which *ζ*_*n*_’s are the eigenvalues of matrix **H** and *Y*_*n*_’s are proportional to the eigenvectors. Also, *c*_*n*_’s are constant coefficients. By comparing (8) and (1), it is found that *F*_*r*_ is the reconstructed array factor and the eigenvectors and eigenvalues of the characteristic matrix are proportional to the excitation currents and the location of the array elements, respectively. After specifying the eigenvalues *ζ*_*n*_, and by comparing Eqs. (1) and (8), the locations of the array elements can be determined as.

9 $$ z_{n} = \frac{{L\alpha_{n} }}{2\pi \lambda } \, \to \, \alpha_{n} = \measuredangle \left( {\frac{{\zeta_{n} }}{{\left| {\zeta_{n} } \right|}}} \right), \, - \pi \le \alpha_{n} \le \pi $$

It is clear that *α*_*n*_’s are the phase of the normalized eigenvalues of the characteristic matrix. After specifying the location of array elements, and because of the savings in the computational cost, the Least Square Method (LSM) is used to determine the excitation currents instead of determining the eigenvectors of the characteristic matrix. To this end, the vector including the samples of the desired array factor and the coefficient matrix **A** are established as follows, in which *P* is the total number of the samples.

10 $$ {\mathbf{B}} = \left[ {\begin{array}{*{20}c} {F_{d} \left( {u_{1} } \right)} & {F_{d} \left( {u_{2} } \right)} & \ldots & {F_{d} \left( {u_{P} } \right)} \\ \end{array} } \right]^{T} $$

11 $$ {\mathbf{A}} = \left[ {\begin{array}{*{20}c} {e^{{ju_{1} z_{1} }} } & {e^{{ju_{1} z_{2} }} } & \cdots & {e^{{ju_{1} z_{N} }} } \\ {e^{{ju_{2} z_{1} }} } & {e^{{ju_{2} z_{2} }} } & \cdots & {e^{{ju_{2} z_{N} }} } \\ \vdots & \vdots & \ddots & \vdots \\ {e^{{ju_{P} z_{1} }} } & {e^{{ju_{P} z_{2} }} } & \cdots & {e^{{ju_{P} z_{N} }} } \\ \end{array} } \right] $$

The value of *P* is determined using the Nyquist sampling rate as^17^.

12 $$ P \approx {{4L} \mathord{\left/ {\vphantom {{4L} \lambda }} \right. \kern-\nulldelimiterspace} \lambda } $$

Therefore, the following system of equations is obtained, in which vector **X** contains the excitation currents.

13 $$ {\mathbf{A}}_{P \times N} {\mathbf{X}}_{N \times 1} = {\mathbf{B}}_{P \times 1} $$

The excitation currents can be calculated using the least square method as follows^23^.

14 $$ {\mathbf{X}} = \left( {{\mathbf{A}}^{T} {\mathbf{A}}} \right)^{ - 1} {\mathbf{A}}^{T} {\mathbf{B}} $$

**Figure 1:**
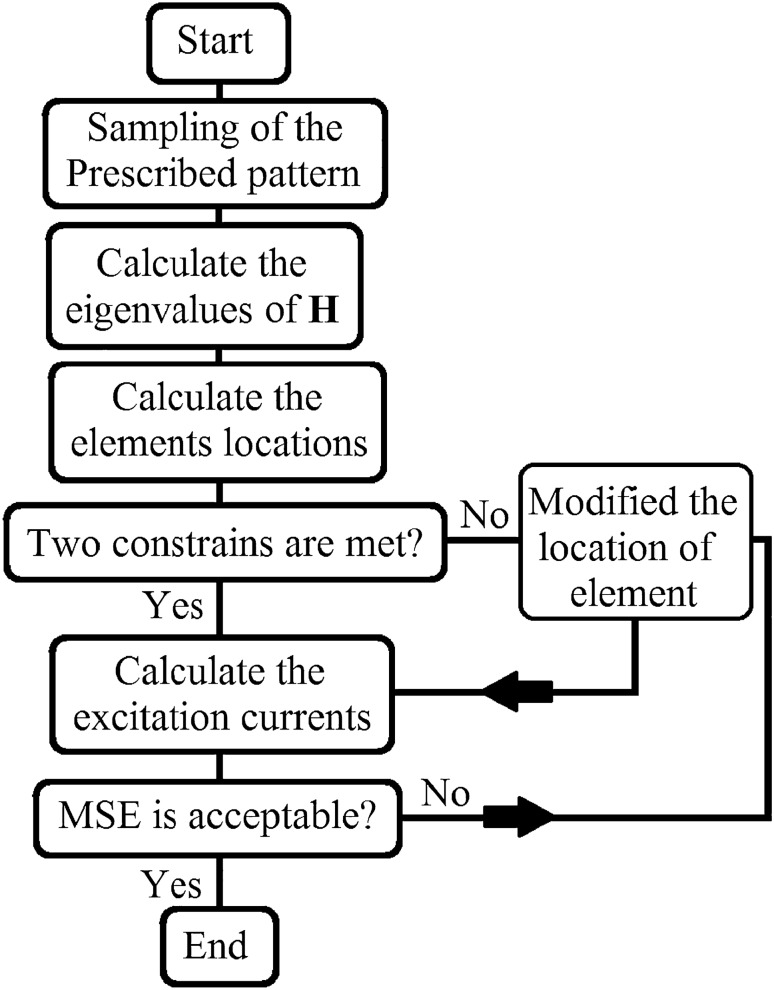
The flowchart of the proposed method.

Our studies show that the location of the elements obtained from Eq. (9) may be unreasonable, increase the mutual coupling, or even cause grating lobes in the radiation pattern. To overcome the problem, we add the following constraints to the problem^21^.

15 $$ d_{0} \le \left| {d_{m} - d_{m \pm 1} } \right| \le \frac{\lambda }{{1 + \left| {\cos \theta_{0} } \right|}} $$

where *d*_*0*_ is the minimum allowable element spacing that controls the mutual coupling, which is determined by the designer. Also, *θ*_*0*_ is the angle at which the radiation pattern is maximum. No grating lobe will appear in the radiation pattern as long as the distance between two adjacent elements is in condition Eq. (15)^21^. Therefore, if the mentioned condition is not met, the location of that element must be modified. This can be done using an iterative process. The flowchart of the proposed process is presented in Fig. 1.

## Reducing the number of array elements

The total number of array elements can be reduced using the Eigen value decomposition (EVD). To this end, it is assumed that a hypothetical matrix **A** with rank *N* can be rearranged using a *Q* × *Q* diagonal matrix **W**, including *Q* non-zero eigenvalues as.

16 $$ {\mathbf{AE}} = {\mathbf{EW}} $$

where **E** is an eigenvector, *Q* is the total number of non-zero eigenvalues. Additionally, if **A** is a full rank matrix (*N* = *Q*), it can be factorized as follows^24^.

17 $$ {\mathbf{A}} = {\mathbf{EWE}}^{ - 1} = \sum\limits_{n = 1}^{N} {\zeta_{n} {\mathbf{g}}_{n} {\mathbf{v}}_{n}^{T} } $$

The above equation shows that **A** can be expressed as the sum of *N* sub-matrices with rank 1. Also, it shows that every sub-matrix is distinctly determined by multiplying two vectors **g**_n_ and **v**_n_ that are orthogonal with each other.

The eigenvalues *ζ*_*n*_ with the very small value can be considered as non-important components of *F*_*d*_ , such as noise, and they can be ignored. The rest of the eigenvalues reflect the important components of *F*_*d*_. It is assumed that the *Q* numbers of eigenvalues correspond to the dominant components of *F*_*d*_. So, The Differential Spectrum Method can be used to calculate of the number of element *Q*^25^. Then, matrix **A**_q_ with the rank *Q* will be determined as the following equation^24^.

18 $$ {\mathbf{A}}_{q} = {\mathbf{EW}}_{q} {\mathbf{E}}^{ - 1} = \sum\limits_{q = 1}^{Q} {\zeta_{q} {\mathbf{g}}_{q} {\mathbf{v}}_{q}^{T} } $$

Also, if the samples of *F*_*d*_ be in a small subspace of rank *N*, a simple Monte Carlo algorithm can also be used to determine the principal components^24^. Also, if the samples of *F*_*d*_ be in a very high dimensional, a similar algorithm can be applied using the sampling method. It is important to note that this technique can also be used for an equally spaced array.

If we denote the number of array elements before and after applying the reduction process by *N* and *Q*, respectively, then the reduction percent *η* can be defined as, in which 0 ≤ *η* ≤ 100.

19 $$ \eta = \frac{N - Q}{N} \times 100 $$

## Results and discussion

In this section, to verify the performance of the proposed method, several practical arrays are investigated, and the obtained results are compared.

### Synthesizing of Kumar and Branner Pattern

**Figure 2:**
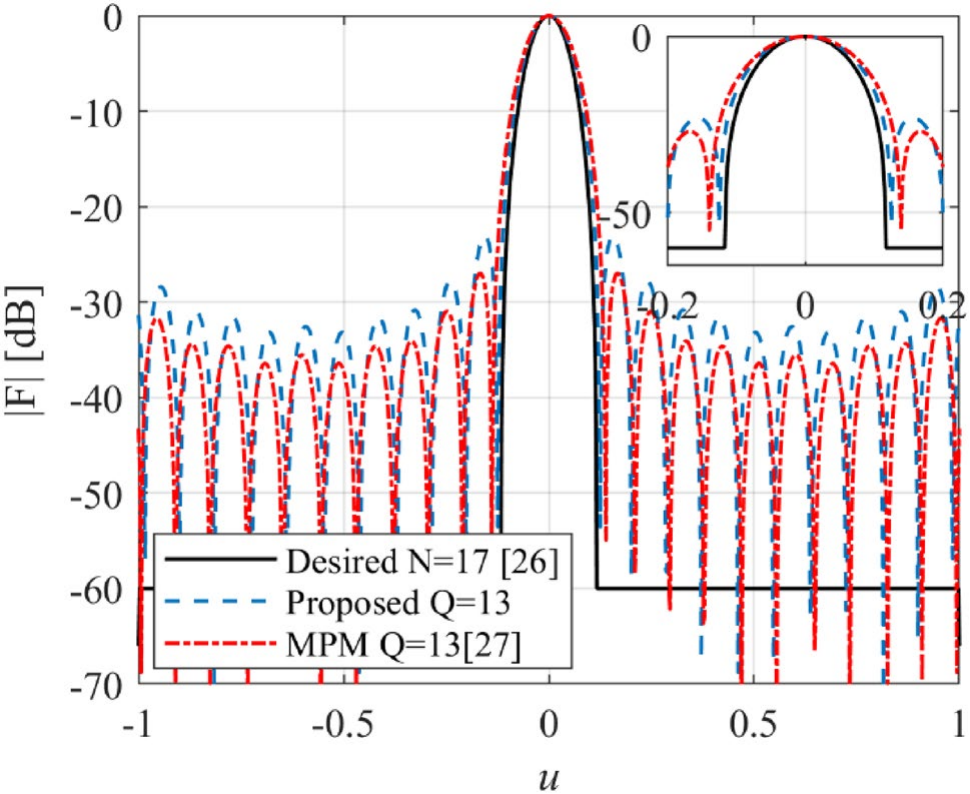
The synthesized results of Kumar and Branner.

**Figure 3:**
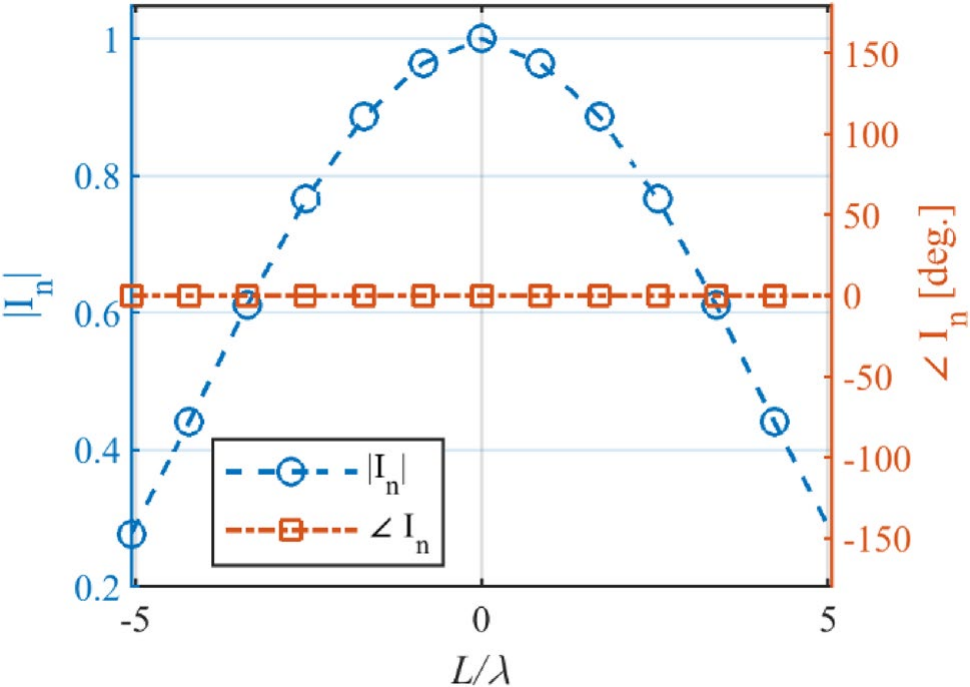
The calculated *I*_*n*_ versus *L*/λ for Kumar and Branner pattern.

In the first example, an array with the prescribed array factor introduced by Kumar and Branner with 17 elements is considered^26^. This array factor is synthesized using the proposed and matrix pencil methods (MPM)^27^. In Fig. 2, the obtained results are compared. The number of array elements for the proposed and MPM methods is lower than the introduced method in^26^. The reduction efficiency of the proposed and MPM methods is at the same level. The accuracy of the MPM methods in the side lobe region is a little higher than the proposed method, but the half-power beam-width of the proposed method is better than MPM. In Fig. 3, the obtained excitation coefficients of the designed array using the proposed method are plotted versus the locations of elements. It can be seen that all excitation currents are real, but those obtained by MPM are complex.

### Synthesizing of flat-top pattern

**Figure 4:**
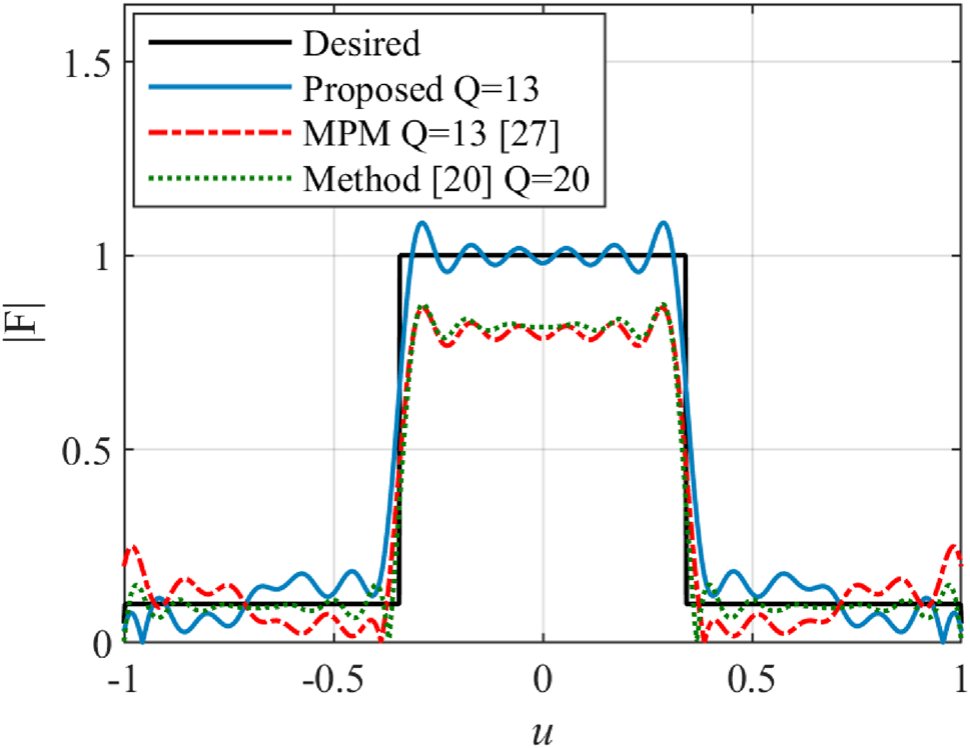
The synthesized results of the flat-top pattern.

**Figure 5:**
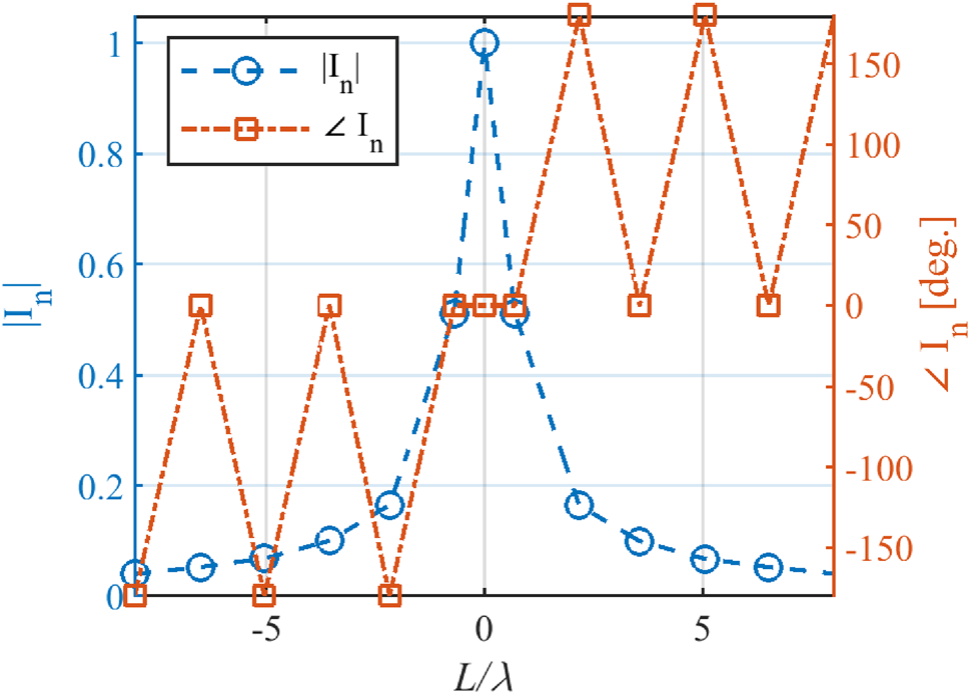
The calculated In versus L/λ for the flat-top pattern.

An array with the flat-top pattern is widely used in communication systems. Since there are several sudden jumps in a flat-top pattern, the synthesizing of it is a serious challenge. In the second example, a flat-top pattern with non-zero values over the interval 70^0^ ≤ *θ* ≤ 110^0^ (-0.342 ≤ *u* ≤ 0.342) is considered. The synthesized result using the proposed, MPM^27^ and the introduced method in^20^ are shown in Fig. 4. The number of array elements for the proposed and MPM methods is lower than the introduced method in^20^. Although the number of array elements of the proposed and MPM method is at the same level, the accuracy of the proposed method is higher than MPM technique. The results of MPM show a difference of about 0.25 in the sector region. The result of the proposed method shows the maximum ripple lower than 0.1 in both the sector and side lobe regions. The phase and magnitude of the calculated excitation currents using the proposed method along the array length are plotted in Fig. 5.

Since the performance of the proposed and MPM method are close together, the comparison between these methods are reported in Table 1. The comparison includes the running time (*t*), mean square error (*MSE*), and the minimum spacing between the two adjacent elements (*MS*/λ). It is seen that the MPM method is very faster than the proposed method because the proposed method is an iterative technique. However, this is not so important with today's powerful computers. The error comparison shows that the accuracy of the introduced method is higher than the MPM method.

**Table 1:** Comparison of the proposed and MPM methods.

Example		Kumar-Branner	Flat-top
*t* (s)	Proposed Method	1.9	1.7
	MPM	7.3e−3	7.0e−3
*MSE*	Proposed Method	0.0010	0.0066
	MPM	0.0018	0.0198
*MS*/λ	Proposed Method	0.84	0.7
	MPM	0.79	0.6

It can also be noted that the proposed method's minimal distance between two adjacent items is greater than the MPM methodology. It means that the proposed technique's mutual coupling for designed arrays is better than the MPM method^17^. Furthermore, Table 2 compares the performance of the proposed method and the other ones available in the literature.

**Table 2:** The performance comparison of the proposed and other methods.

	This work	^4^	^18^	^19^	^20^
Complexity	High	High	High	Middle	Middle
Accuracy	High	Middle	Middle	Middle	High
Reduction capability	Yes	Yes	No	Yes	Yes
Mutual coupling controlling	Yes	Yes	No	No	No

### Practical array simulation

**Figure 6:**
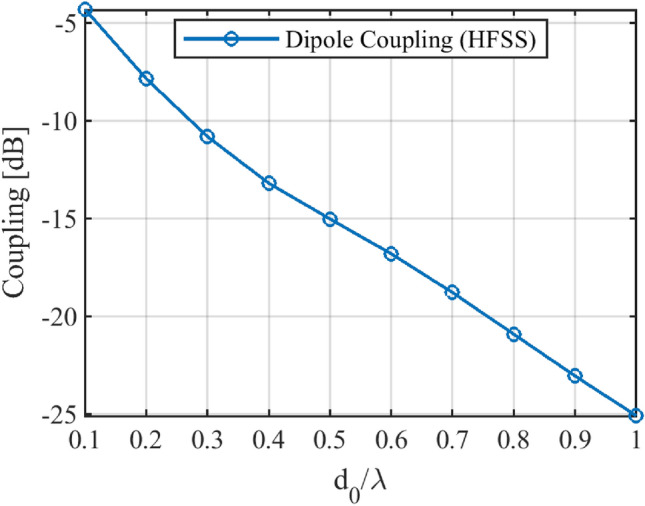
Coupling between two dipoles antenna versus the distance between.

In this section, to assess the performance of the suggested method, a practical array made of half-wavelength dipole antennas is simulated by HFSS software. To this end, the desired array factor with Taylor distribution^28^ with the first side lobe of about 20 dB is considered. First, the value of *d*_*0*_ must be determined. Figure 6 shows the coupling between two dipoles antenna versus the distance between them. It is desirable that the coupling between the antennas be better than − 16 dB. According to this figure, the value of *d*_*0*_ in Eq. (15) should be selected as equal to 0.58λ.

**Figure 7:**
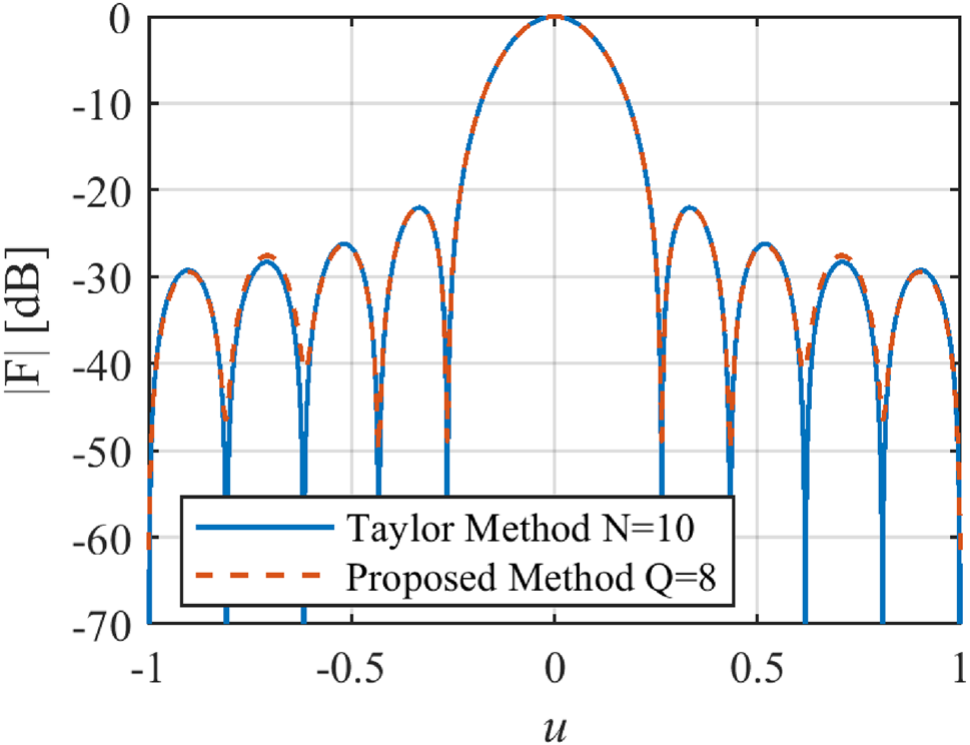
The synthesized results of the Taylor pattern.

**Figure 8:**
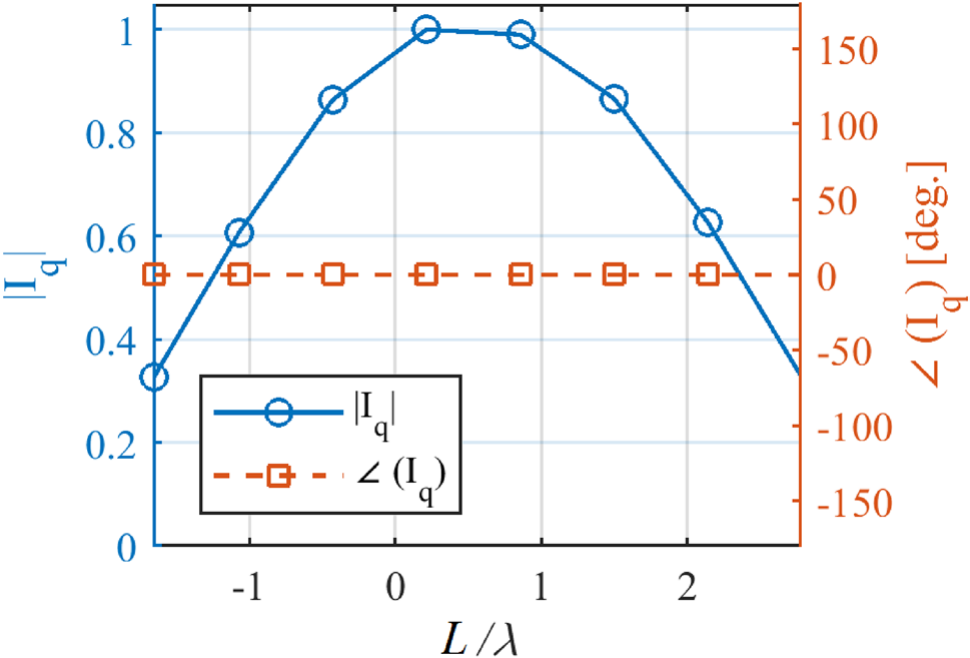
The calculated In versus L/λ for the Taylor pattern.

As shown in Fig. 7, by applying the proposed method for the required characteristics, the prescribed array factor is reconstructed by only *Q* = 8 elements, but the number of elements required to reconstruct the desired array factor by the Taylor method^28^ is *N* = 10. The phase and magnitude of the calculated *I*_*n*_ using the proposed method along the array length are plotted in Fig. 8.

**Figure 9:**
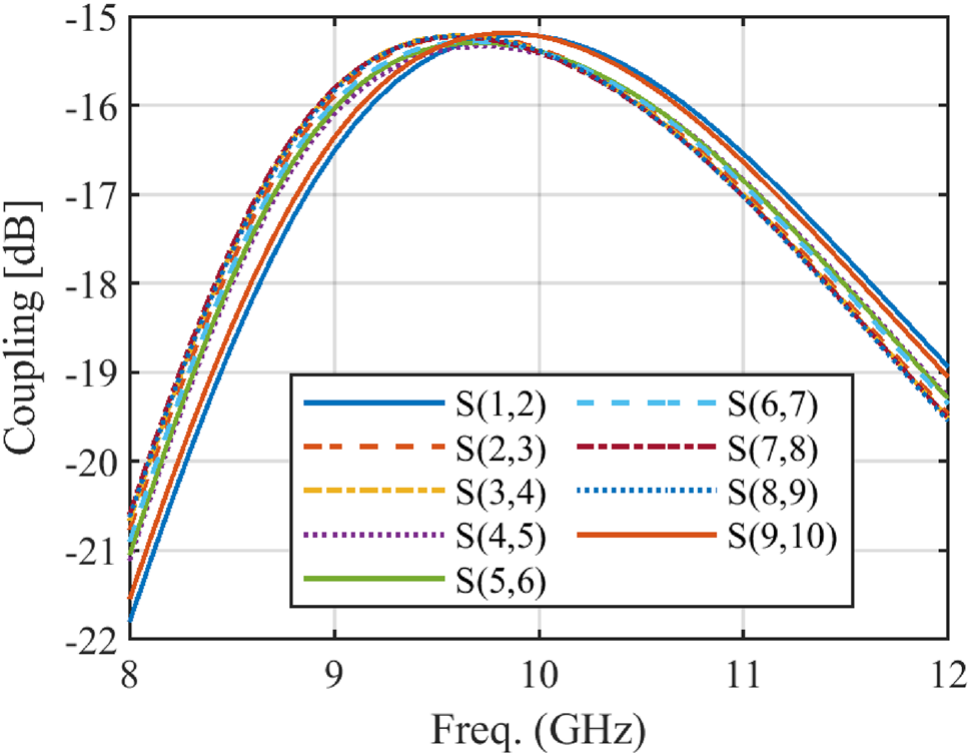
The simulated mutual coupling between different elements of the designed array with Taylor method.

**Figure 10:**
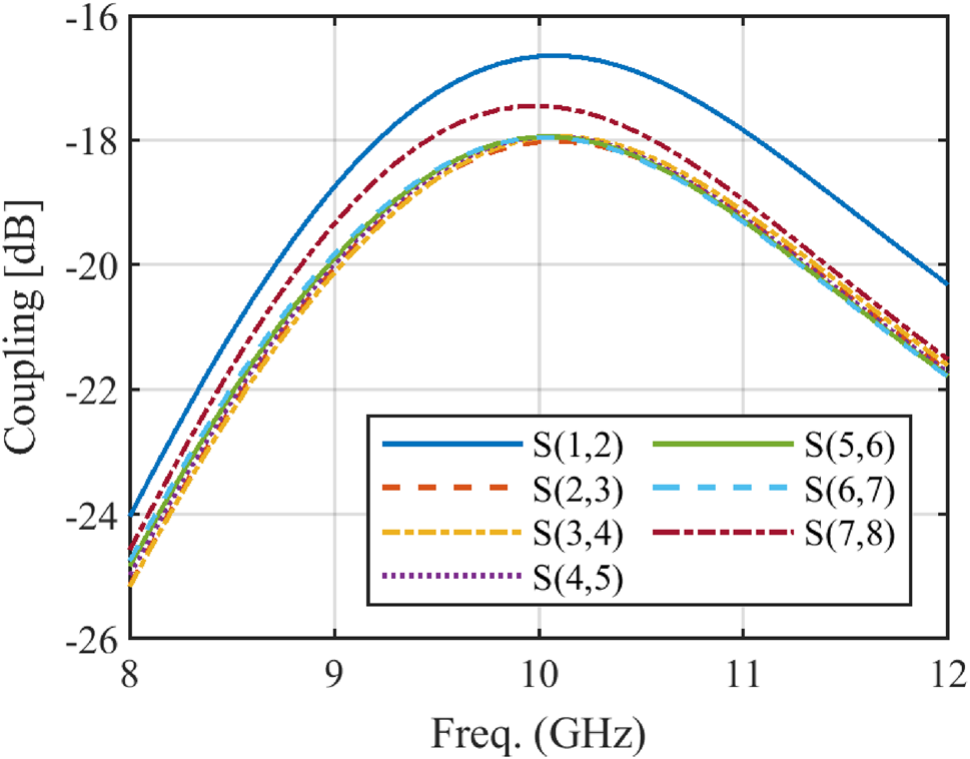
The simulated mutual coupling between different elements of the designed array with the proposed method.

The designed array is implemented in HFSS, and mutual coupling is taken into account fully. Figure 9 and Fig. 10 depict the simulation results of mutual coupling between two adjacent elements for both arrays implemented in HFSS. The maximum coupling between the elements is approximately − 16.8 dB, compared to − 15 dB for the Taylor method. In comparison to the Taylor method, the proposed method improves the mutual coupling for the designed array by at least 1.8 dB, as expected. It can be concluded that the improvement is due to the increase in the distance between the elements of the array.

**Figure 11:**
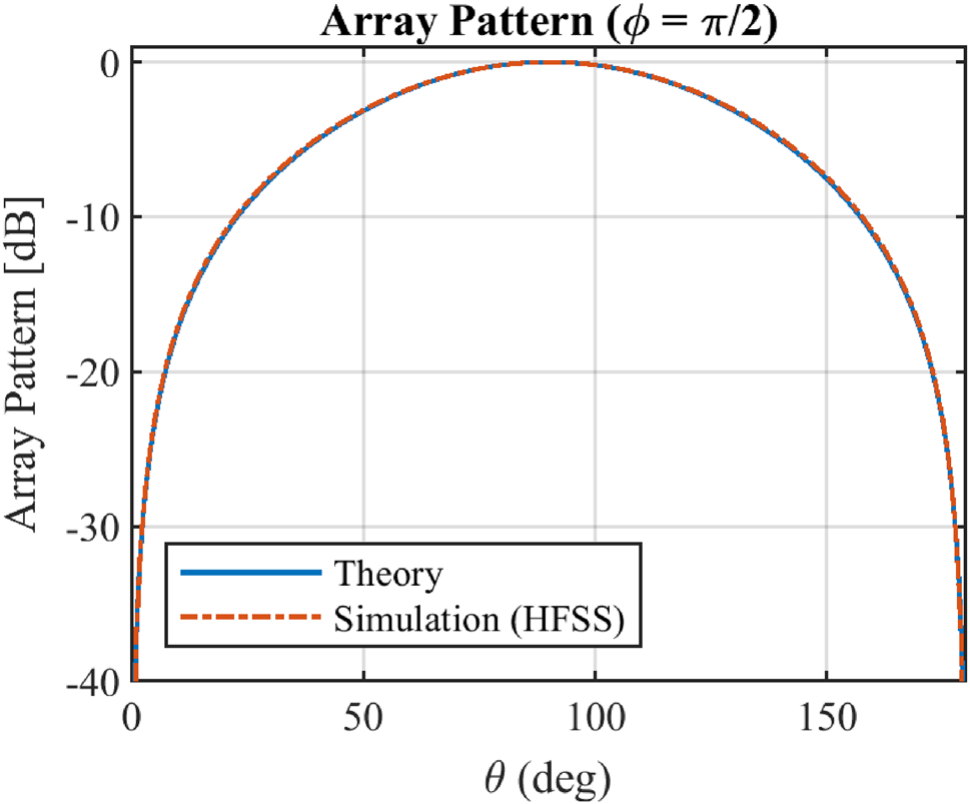
The total radiation pattern of array at φ = π/2 including the mutual coupling effect considered in simulation study by HFSS.

**Figure 12:**
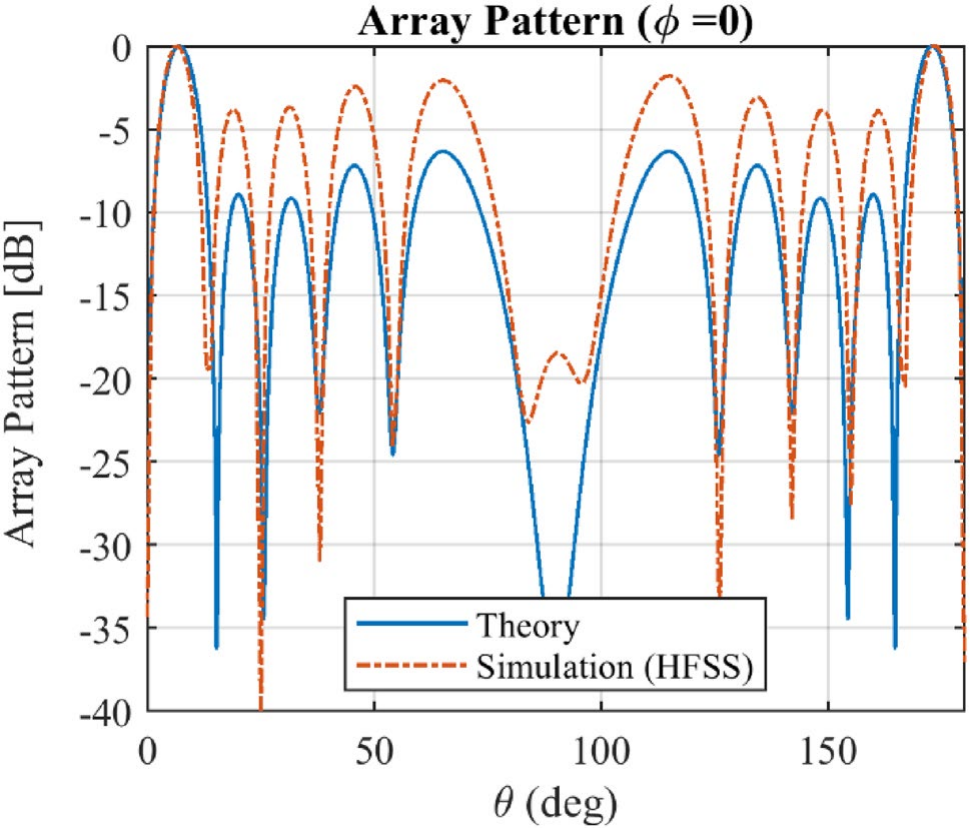
The total radiation pattern of array at *φ* = 0 including the mutual coupling effect considered in simulation study by HFSS.

Figure 11 and Fig. 12 show the total radiation pattern of the implemented array in HFSS with *Q* = 8 elements including element and array factor patterns at standard planes of *φ* = 0 and *φ* = π/2. Since in the simulation process, the array elements are arranged along the x-axis, the array factor in *φ* = π/2 has a constant value. It is seen that the simulated and theoretical results for the array pattern in plane *φ* = π/2 are agreed very well, but for *φ* = 0, there is a deviation between the simulated and theoretical ones. It should be noted that the proposed method is based on the array factor synthesizing using isotropic elements. The element pattern and mutual coupling effect should be considered in real-world problems.

## Conclusion

In this paper, a new method is proposed for the synthesis of the radiation pattern of an unequally-spaced array. This method is based on the fractional Fourier series and eigenvalue decomposition. In this method, the location of the array elements is calculated by considering the mutual coupling effect. The proposed method can aid in the reduction of array elements. To verify the performance of the proposed method, several practical examples with various properties were investigated, and a comprehensive discussion and comparison with the other introduced methods is presented. The obtained results show that the introduced method has a good performance in reducing the mutual coupling and number of array elements, while still retaining a good ability to approximate the desired pattern fairly faithfully, especially for beam-shaped patterns.
